# The Role of Neutrophils in Pregnancy, Term and Preterm Labour

**DOI:** 10.3390/life12101512

**Published:** 2022-09-28

**Authors:** Belen Gimeno-Molina, Ingrid Muller, Pascale Kropf, Lynne Sykes

**Affiliations:** 1 Imperial College Parturition Research Group, Department of Metabolism, Digestion and Reproduction, Imperial College London, London W12 0HS, UK; 2 March of Dimes European Prematurity Research Centre, Imperial College London, London W12 0HS, UK; 3 Department of Infectious Diseases, Imperial College London, London W2 1NY, UK; 4 The Parasol Foundation Centre for Women’s Health and Cancer Research, St. Mary’s Hospital, Imperial College Healthcare NHS Trust, London W2 1NY, UK

**Keywords:** neutrophils, pregnancy, term labour, premature labour, vaginal microbiota, inflammation

## Abstract

Neutrophils are surveillance cells, and the first to react and migrate to sites of inflammation and infection following a chemotactic gradient. Neutrophils play a key role in both sterile inflammation and infection, performing a wide variety of effector functions such as degranulation, phagocytosis, ROS production and release of neutrophil extracellular traps (NETs). Healthy term labour requires a sterile pro-inflammatory process, whereas one of the most common causes of spontaneous preterm birth is microbial driven. Peripheral neutrophilia has long been described during pregnancy, and evidence exists demonstrating neutrophils infiltrating the cervix, uterus and foetal membranes during both term and preterm deliveries. Their presence supports a role in tissue remodelling via their effector functions. In this review, we describe the effector functions of neutrophils. We summarise the evidence to support their role in healthy pregnancy and labour and describe their potential contribution to microbial driven preterm birth.

## 1. Neutrophils and Their Effector Functions

Neutrophils, also known as granulocytes, are a type of polymorphonuclear innate immune cell, and comprise 50–70% of circulating leukocytes. As part of the innate immune system, they are the first line of defence during infection, however they also play a role in sterile inflammation and tumorigenesis [1]. Neutrophils are typically characterized by a distinctive multi-lobulated nucleus and the presence of granules in their cytoplasm. They originate from the myeloid progenitor cells located within the bone marrow and extramedullary sites including the spleen. The process that leads to the production of neutrophils is known as granulopoiesis, with differentiation mainly driven by IL-17, G-CSF, and IL-1β. Neutrophils are also characterised as a heterogeneous cell type, with various subpopulations depending on temporal and anatomical factors [2]. They express different markers on their surfaces that change according to the maturation status, allowing them to exhibit high plasticity to respond depending on conditions of health and disease [3,4,5].

**Figure 1:**
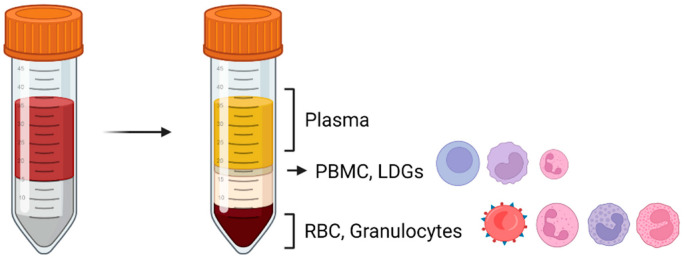
Isolation of immune cells. Blood fractioning and separation by density centrifugation. The top layer contains plasma. The dense halo contains several leukocyte populations: mainly PBMC (peripheral blood mononuclear cells) including lymphocytes and monocytes. This fraction also contains low-density neutrophils. The bottom layer contains red blood cells (RBC, erythrocytes) and granulocytes: neutrophils, basophils, and eosinophils. Image created with Biorender.com.

**Figure 2:**
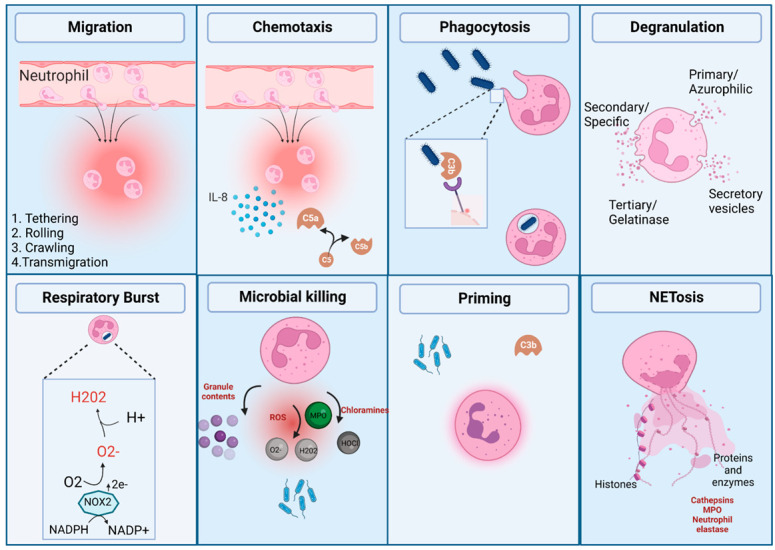
Neutrophil effector functions. Neutrophils from the circulation are able to migrate into tissue via a chemotactic gradient. At sites of infection and inflammation they perform several effector functions: phagocytosis, degranulation, production of ROS (reactive oxygen species) via respiratory burst, microbial killing, and release of neutrophil extracellular traps (NETs). Neutrophil activation is enhanced if previously primed. Created with BioRender.com.

There are two major distinct subpopulations: normal density and low-density neutrophils, that can be differentiated and retrieved via density gradient separation. Normal density neutrophils will precipitate with other polymorphonuclear cells, whereas low-density neutrophils (low density granulocytes (LDGs) co-purify with PBMCs (peripheral blood mononuclear cells) (Figure 1). Less is understood on the functions of LDGs, however they are known to exhibit both immunosuppressive and pro-inflammatory properties, and are abundant in inflammatory conditions that are associated with relative immunosuppression such as HIV [6], tuberculosis [7], visceral leishmaniasis [8] and systemic lupus erythematosus (SLE) [9]. Pregnancy and labour, both term and preterm are associated with an increase in circulating normal density and low-density neutrophils [10,11,12], suggesting they play a functional role. Circulating neutrophils migrate into tissue in response to certain triggers. In this review, we summarise the effector functions of neutrophils, and their role in both the physiological and pathological processes relating to pregnancy, term and preterm labour. The effector functions of neutrophils are summarised below and are illustrated in Figure 2.

### 1.1. Migration and Chemotaxis

The process of migration involves several steps: tethering, rolling, adhesion, crawling and transmigration, reviewed by Kolazkowska et al. [13]. The mechanism of tethering and adhesion is highly dependent on the expression and interaction between selectins and integrins, as well as the presence of cytokines, chemoattractants and/or growth factors secreted by activated resident sentinel leukocytes. Neutrophil motility is enhanced by the presence of chemoattractants that can direct and organise the migration towards the target along a chemotactic gradient. Chemoattractants include chemokines (IL-8 and RANTES), complement anaphylatoxins (C3a and C5a), formyl peptides (e.g., N-formylated oligopeptides) and chemotactic lipids (e.g., leukotriene B) [14].

### 1.2. Phagocytosis and Opsonisation

Neutrophils phagocytose (from Greek *phagein* “eat” and *kytos* “cell”) by engulfing particles or microorganisms. The mechanism involves the recognition and ingestion via direct recognition of target or opsonins by neutrophil receptors. Phagocytosis is more effective when bacteria are opsonised and coated by opsonins like immunoglobulins or complement proteins. Opsonising antibodies and the complement protein C3b are recognised by surface cell Fc and C3 receptors, respectively. When the opsonised particle attaches to the surface, the membranes invaginate enclosing the microorganism within an internal vesicle. Other opsonins include fibronectin, lipopolysaccharide-binding protein (LBP), thrombospondin, lung surfactant protein A, and conglutin [15].

### 1.3. The Role of the Complement System in Enhancing Neutrophil Phagocytosis

The complement cascade can be activated via 3 different pathways (the classical, the alternative and the lectin pathway), all converging on the activation of C3 convertase, thereby defragmenting C3 to C3a and C3b [16]. Activation of C5 convertase leads to the release of C5a from C5 and formation of the membrane attack complex further down the cascade. The complement proteins C1q, C3b, C4b and iC3b can all bind to pathogens. Recognition of bacteria via immunoglobulins (IgM and IgG) will also activate the complement cascade via the classical pathway, leading to an amplification of complement deposition [15]. Among a variety of membrane surface receptors, neutrophils express Fc-receptors that will recognise Ig-coated pathogens [17] as well as Complement Receptor 1 (CR1, CD35) and Complement Receptor 3 (CR3, CD11b/CD18 or MAC-1) that will bind with different affinity to pathogens opsonised by C1q, C3b, C4b and iC3b [15]. Therefore, interaction between the complement proteins and neutrophils serves to co-locate pathogens with neutrophils, thereby enhancing phagocytosis, but also chemotaxis via the C5a receptor (C5AR1, CD88) [17].

### 1.4. Neutrophil Degranulation

Another effector function of activated neutrophils is their ability to degranulate and release contents required for direct microbial killing. Following stimulation by chemoattractants or opsonised particles, neutrophils release the contents of their cytoplasmatic granules. Neutrophils acquire their granules during the maturation process in the bone marrow. The process of degranulation requires that the membranes of the granule fuses with another cellular membrane; it can be either the phagocytic vesicle (intracellular degranulation) or the plasma membrane (extracellular degranulation) [18]. Neutrophils contain four different types of granules or vesicles (see Table 1 for summary). Most of the contents are microbicidal agents, therefore implicated in the killing and digestion of ingested particles [19,20]. Primary granules (also known as azurophilic because they stain with azure dye) are the most toxic and are peroxidase positive. Primary granules contain lysozymes, defensins, myeloperoxidases (MPO), proteinases, neutrophil elastase (NE) and sialidase. In contrast, secondary and tertiary granules are peroxidase negative, and overlap certain contents such as lysozymes, matrix metalloproteinases and the C3b receptor. The secondary granules usually discharge their content outside the cell. Finally, secretory vesicles contain plasma and albumin is the most typical marker. In addition to resulting in pathogen cell death, the release of the granule contents will cause collateral damage to the surrounding tissues and prolong the inflammatory response.

Upon activation, neutrophils express genes of pro-inflammatory and pro-labour mediators such as cytokines, chemokines, and cyclooxygenase-2 (COX-2). Cyclooxygenase (COX, PGH synthase) is an enzyme that catalyses the metabolic conversion of arachidonic acid to prostaglandins (PGs) and thromboxanes (TXs). There are two isoforms of COX: COX-1 and COX-2, the latter being constantly expressed at basal levels and further induced in cases of inflammation. Human neutrophils express COX-2 resulting in the production of PGE2. The activity of COX-2 is enhanced by cytokines and factors such as TNF-α and LPS [21,22].

### 1.5. Respiratory Burst

Upon activation, neutrophils can release reactive oxygen species (ROS) during a respiratory burst. This involves the active participation of the enzyme NADPH oxidase (also known as NOX2 in humans), as well as myeloperoxidase. These enzymes are located in the phagolysosome membranes of neutrophils [23]. Secretion of cytochrome (one of the components of the electron transport chain) catalyses the one electron reduction in oxygen to superoxide anions. Superoxide anions are very reactive, being able to spontaneously recombine with other molecules, producing reactive free radicals. As a result, releasing of reactive oxygen species (ROS), superoxide anion (O_2_^−^) and hydrogen peroxide (H_2_O_2_) can lead to direct microbial killing [18].

### 1.6. NETosis

Finally, another impressive defence mechanism of neutrophils is their ability to release Neutrophil Extracellular traps (NETs), in which neutrophils can expel their contents to the exterior, including DNA chromatin and histones, as well as granule proteins (including MPO, NE, azurocidin, proteinase 3, cathelicidin), creating a very complex extracellular structure that can, as per its name, entrap and finally kill bacteria and other microorganisms [24].

### 1.7. The Role of Neutrophils in Sterile Inflammation

The innate immune system is activated through signalling via pattern recognition receptors (PRRs), as they recognise pathogen-associated molecular patterns (PAMPs). Examples of PRRs are the membrane receptors Toll-like receptors (TLR), and the intracellular receptors NOD-like receptors (NLRs). After recognition of a ligand/protein this will activate internal downstream signalling pathways, typically involving NF-κβ and MAP kinases, resulting in the active participation of leukocytes such as neutrophils. Inflammation can occur in the absence of infection, described as “sterile inflammation”. PRRs in this case include damage-associated molecular patterns (DAMPs) and will recognise endogenous molecules or signals, ultimately triggering the same downstream pathways [25]. Important mediators of sterile inflammation and recruitment of neutrophils are IL-1 and IL-33 [25,26,27]. DAMPs linked with neutrophil recruitment include activation of the inflammasome leading to IL-1β production, as well as mitochondrial activity, since formation of peptides resembles formyl peptides [28,29].

Sterile inflammation is typical of the process seen in wound repair, as well as autoimmune conditions. It is not surprising that neutrophils have long been implicated in sterile inflammation, since one of the roles of neutrophils is clearance of dead cells and cell debris. The release of proteases degrades the extracellular matrix (ECM) to allow tissue remodelling; and release other factors such as prostaglandins will promote vessel growth [25,29].

## 2. Neutrophils during Healthy Pregnancy

Pregnancy involves a complex immunological state that requires a balance between tolerance and immunosuppression to allow growth of the semi-allogeneic foetus, whilst maintaining an effective defence mechanism that can protect against infection [30]. There is acknowledgement of a highly active and regulated immune response with shifts between predominance of an anti-inflammatory and pro-inflammatory immune milieu, depending on gestational timepoint and anatomical location [31,32]. In the most simplistic description, the first trimester is described as a pro-inflammatory phase, to enable implantation. The second phase resembles an anti-inflammatory phase, where a shift in cytokine bias towards T helper 2 phenotype is thought to contribute to foetal growth. Thirdly, a pro-inflammatory switch is seen in preparation for labour, with influx of immune cells into gestational tissue. Recent signatures from peripheral blood immune cells also support a systemic immune clock for human pregnancy [33].

### 2.1. Peripheral Blood Neutrophils in Healthy Pregnancy

There is an increase in white blood cell counts in pregnancy, especially towards the end; and a further increase at the time of labour [34]. This increase in leukocyte counts is mainly driven by an increase in peripheral granulocyte numbers [35], with several studies confirming that pregnancy is associated with a mild neutrophilia [36]. The immunophenotype of maternal peripheral neutrophils has been shown to reflect increased activation during pregnancy compared to non-pregnant women. Increased capacity for phagocytosis [37,38], increased ROS production [4,39,40], and arginase metabolism [41] have all been demonstrated in peripheral blood neutrophils of pregnant women compared to non-pregnant controls. Furthermore, response to common pathogens associated with an infectious aetiology of preterm birth is heightened in neutrophils taken from pregnant women compared to non-pregnant controls [42].

We have previously also demonstrated that a subtype of neutrophils, LDGs, are present in the peripheral blood of pregnant women. These cells have enhanced arginase expression, and we have shown that this contributes to T cell hypo-responsiveness, which may contribute to the relative immunosuppression seen in pregnancy [43]. Furthermore, we have shown an increase in circulating LDGs in pregnancy, compared to non-pregnant controls, with increased expression of CD15 and CD16 [44]. Furthermore, we have reported that LDGs taken from maternal peripheral blood at term, preceding the onset of labour, are more activated than NDGs with increased CD15, CD66b and CD63 expression, consistent with exhibiting a pro-inflammatory phenotype [45].

The mechanism and rationale for the increase in activity of peripheral blood neutrophils, especially LDGs is not fully understood. Some explanation for the increase in circulating cells comes from a combined effect of increased bone marrow production, consistent with an increase in circulating immature granulocytes [46], and impaired neutrophil apoptosis [10]. Increased concentrations and activation are likely to provide an important pregnancy related mechanism for enhancing the innate immune response, whilst compensating for the attenuation of the cell mediated immune response [47]. It has also been postulated that an enhanced systemic inflammatory response in pregnancy could be mediated by particulate debris or soluble products derived from the placenta [48].

### 2.2. Neutrophils at the Maternal–Foetal Interface in Healthy Pregnancy

Neutrophils are detected in the decidua of healthy human pregnancy from as early as the first trimester. It is thought that decidual neutrophils play a key role in tissue remodelling and placental vascularization-like spiral artery remodelling [49]. Detection of neutrophils in amniotic fluid is also seen and increases with advancing gestational age [50]. Their presence is likely to ensure protection against any invading pathogens in a highly sterile environment, to protect the growing foetus. In contrast, there is sparse distribution of neutrophils in myometrium and the cervix prior to the onset of labour [51,52].

## 3. The Role of Neutrophils in Human Term Labour

**Figure 3:**
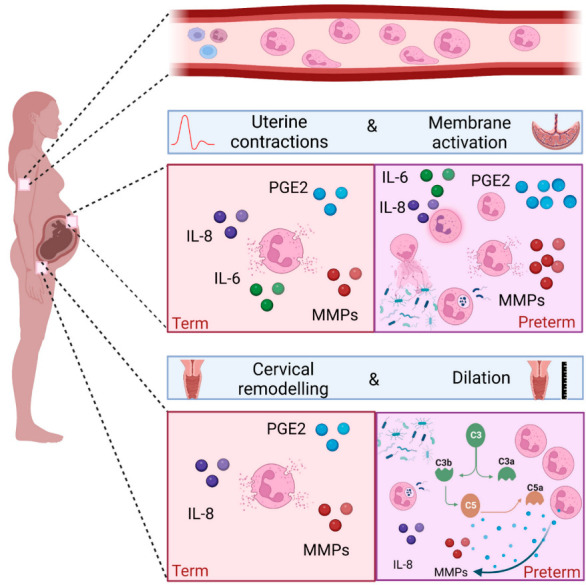
The role of neutrophils in term birth and microbial-driven preterm birth. The number of circulating peripheral blood neutrophils increase during healthy pregnancy. Neutrophils can migrate into tissue following a chemotactic gradient. Term labour (left) is associated with infiltration of neutrophils in the lower uterine segment, foetal membranes and cervix. Their detection is accompanied by an increase in cytokines such as IL-8 and MMPs participating in the degradation of the extracellular matrix, therefore contributing to tissue remodelling. Neutrophils are also a source of COX-2 and PGE2, therefore can contribute to uterine contractility membrane activation and cervical dilation. In cases of infection (right), neutrophils are detected in foetal membranes and amniotic fluid, along with an increase in pro-inflammatory mediators. Ex vivo experiments using neutrophils demonstrate their capacity to perform phagocytosis, NETosis and release of pro-inflammatory mediators. Finally, an adverse vaginal microbial composition is associated with an increase in cervical neutrophils, inflammatory mediators and complement activation. Image created with Biorender.com.

Successful pregnancy requires uterine quiescence, the foetus to be contained in the amniotic sac, and the cervix to remain long and closed [53,54]. The process of parturition requires the onset of uterine contractions, rupture of the foetal membranes, and dilatation of the cervix. This ultimately leads to delivery of the foetus and the placenta, and subsequent involution of the uterus and tissue repair of the cervix. These events are driven by mechanical, inflammatory, and endocrine processes, whose regulation are complex and interlinked [55,56]. These key processes depend on pro-labour mediators such as COX-2, prostaglandins, matrix metalloproteinases and pro-inflammatory cytokines such as IL-8 [57,58,59], all of which can be produced by neutrophils [60,61,62,63]. Despite the triggers of term labour being poorly understood, it is associated with both systemic and local sterile inflammation, with much evidence to support a functional role for neutrophils. Figure 3 illustrates the physiological processes and their anatomical locations where neutrophils are considered to play a key role in term birth.

### 3.1. Peripheral Blood Neutrophils and Term Labour

The onset of labour is consistently associated with neutrophilia [12,64]. Furthermore, peripheral blood neutrophils taken from women in labour show signs of increased activation compared to women not in established labour [11]. In addition, an increase in markers of migration such as CD11a/b and CD62L is seen in vivo, and in vitro studies have confirmed the increased migratory capacity of neutrophils taken from women in labour [12,64]. Taken together, this implies they are primed for migration to sites such as the maternal–foetal interface and the cervix to play a role in the processes required for labour, such as tissue remodelling.

### 3.2. Neutrophils and the Uterus in Term Labour

Neutrophil infiltration of the myometrium is seen during labour [52,61] and unsurprisingly this coincides with an increase in cell adhesion molecule expression to aid transmigration [12]. Expression of the chemoattractant *CXCL8* mRNA was found to be higher in myometrium of women at term during labour compared to term not in labour, which may explain in part the increased abundance of neutrophils during labour [65]. Consistent with this, a parallel increase in IL-8 concentrations and neutrophil counts are seen in the lower uterine segment in women who are in active labour. This is also associated with increased concentrations of matrix metalloproteinases 8 and 9 [66]. Transcriptomic studies have shown increased gene expression in the myometrium of labouring women of 110 genes, clustered into groups reflecting acute inflammation and immune cell trafficking [65]. Immunohistochemistry staining of the cytokines IL-6, IL-8 and TNF-α appear to be restricted to leukocytes in myometrial biopsies, suggesting immune cell infiltration is the predominant source of local myometrial inflammation [62].

### 3.3. Neutrophils and the Foetal Membranes in Term Labour

Leukocytes, including neutrophils, are known to infiltrate foetal membranes at the time of term labour [61,62], and this is accompanied by an increase in the concentrations of pro-inflammatory cytokines such as IL-1β, IL-6, IL-8 [61,62], and pro-labour mediators such as COX-2 and PGE2 synthases [67,68].

### 3.4. Neutrophils and Cervical Remodelling in Term Labour

To facilitate vaginal delivery, cervical dilation is one of the processes that occurs during labour. It is a complex process involving softening, effacement and ripening of the cervix. Biochemical changes are required, including a decline in collagen synthesis, an increase in collagenase activity, local immune cell infiltration, and increased concentrations of cytokines and prostaglandins [69]. Several studies have demonstrated cervical infiltration of neutrophils from biopsies taken at the time of labour [52,61,66,70]. Furthermore, the content of the cervical mucous plug is rich in neutrophils [71]. There are conflicting opinions about the importance of cervical neutrophils in causing the cervical changes required for labour, since not all human and murine studies have demonstrated increased neutrophil density in the presence of cervical ripening [52,72]. Despite this, the key mediators of cervical ripening are products of neutrophil activation and have been shown to co-localise in association with neutrophil infiltration. IL-8, a pro-inflammatory cytokine that is a chemoattractant and activates neutrophils, is increased in the cervix at the time of labour [52,73]. Matrix metalloproteinases, which are contents of neutrophil granules, are also increased in the cervix at the time of labour [74] and contribute to collagen degradation during cervical ripening [66,73,75].

## 4. The Role of Neutrophils in Preterm Labour

Preterm birth (PTB) is defined as a birth occurring before 37 completed weeks of pregnancy and can be further classified as extremely preterm (<28 weeks), very preterm (28–32 weeks), and moderate to late preterm (32–36 weeks). There are 15 million babies born preterm each year, with global rates averaging 10%, although significant variations (5–18%) exist depending on geographical location [76]. PTB is the biggest cause of childhood mortality under the age of 5, with morbidity and mortality increasing with decreasing gestational age at delivery [77]. Roughly two thirds of births are spontaneous, with women presenting either with preterm prelabour rupture of membranes (PPROM), or with uterine contractions and cervical dilation. The causes of preterm birth are multifactorial, but the most common causal factors are infection and/or inflammation. Extreme and very preterm birth are most likely to have evidence of infection and/or inflammation, and babies born with evidence of foetal inflammatory response have a worse prognosis for any given gestational age.

Since neutrophils play a major role in infection and inflammation, they are also likely to play a key role in infection and inflammation in the context of preterm birth. The most common source of intrauterine infection is ascending pathogenic microbes from the cervical-vaginal interface [78]. The presence of intrauterine infection is associated with neutrophil infiltration and pro-inflammatory cytokine production [79]. PPROM often presents with or leads to clinical signs of chorioamnionitis, with neutrophil invasion of the chorioamnion being the hallmark of histological chorioamnionitis [80]. Evidence also exists to support the role of neutrophils in driving local inflammation at the cervical-vaginal interface in women who deliver preterm [7,81,82,83].

Inflammation in the absence of infection, also referred to as sterile inflammation, has also been widely reported in the context of preterm birth [84,85,86]. DAMPs, also known as alarmins, are endogenous molecules that send a danger message as part of a response to inflammation. Alarmins that have been commonly associated with preterm labour include high mobility group box 1 (HMGB1), IL-1α, and cell free DNA. HMGB1 [87,88] and IL-1 α [89,90] concentrations are higher in amniotic fluid of women who have evidence of sterile inflammation and deliver preterm. Cell free DNA activates Toll-like receptor 9 (TLR-9), and animal models support the concept of cell free DNA leading to preterm delivery via leukocyte migration and inflammation at the maternal–foetal interface [91,92].

The more recent ability to interrogate the role of microbial communities in a culture independent way by utilising next generation sequencing may divert us away from the concept of sterile inflammation alone in driving preterm birth. Figure 3 illustrates the pathological processes and their anatomical locations where neutrophils are considered to play a key role in preterm birth.

### 4.1. The Role of the Vaginal Microbiome and Ascending Infection/Inflammation

The vaginal microbial community is typically dominated by *Lactobacillus* spp. in women of reproductive age. Changes to the homeostatic state can lead to dysbiosis, with expansion of pathogenic microorganisms commonly associated with bacterial vaginosis, such as *Gardnerella vaginalis*. Healthy pregnancy is associated with the dominance of *Lactobacillus* spp., and increased stability in the vaginal microbial composition, due to the influence of increased concentrations of oestrogen [93,94,95]. The last decade has led to a vast increase in our understanding of which microbial communities are associated with protection against preterm birth, and which are associated with increased risk. Consistently, it has been shown that vaginal microbial communities dominated by *Lactobacillus crispatus* are associated with a lower risk of preterm birth, whereas a dominance of *Lactobacillus iners,* and bacterial vaginosis (BV) associated taxa such as *Gardnerella*, *Ureaplasma*, *Prevotella* and *Mycoplasma* is associated with a higher risk [96,97].

The vaginal microbiome at the time of rupture of membranes is also highly diverse, with general reduction in *Lactobacillus* spp. and increased richness; with typical predominance of taxa from non-lactobacilli and heterogenous communities (community state type IV, (CST IV)), such as *G. vaginalis, Meghasphaera, Prevotella,* etc. [98,99,100,101].

Evidence is emerging supporting the role of microbial-induced inflammation in modifying risk, with higher cervical-vaginal concentrations of cytokines such as IL-8, IL-6 and IL-1β and complement proteins C3b, C5 and C5a seen in association with in these high-risk vaginal microbial signatures [102,103,104]. It is plausible, that the predominant leukocyte influencing the local immune milieu is the neutrophil, with early studies beginning to support this [105,106,107].

### 4.2. Peripheral Blood Neutrophils and Preterm Labour and PPROM

Products of the microbiota can be translocated into the circulation, priming and enhancing neutrophil’s function [108]. The use of peripheral blood neutrophil concentrations as a predictor of preterm birth has been explored in women presenting in threatened preterm labour and PPROM, using the neutrophil-to-lymphocyte ratio (NLR) and total neutrophil counts [109,110]. Several studies show an increase in neutrophil counts and the NLR in women who subsequently deliver preterm [12,111]. Peripheral blood neutrophils exhibit a more activated immunophenotype in women who deliver preterm. Gervasi et al. collected peripheral blood from women who had a healthy pregnancy and subsequent term labour and compared the granulocyte phenotype with women who delivered preterm. Using flow cytometry, they identified that granulocytes expressed higher levels of CD11b, CD15 and CD66 in women who delivered preterm [112]. Similarly, Yuan and colleagues also assessed the mean fluorescence intensity (MFI) of cell surface activation markers and adhesion molecules; CD11a, CD11b, CD62L were more highly expressed in women who delivered preterm compared to term [12]. In vitro assays demonstrate that neutrophils are the leukocytes with the greatest capacity to migrate, and that migration is highest in neutrophils taken from women with PPROM or in preterm labour, compared to women not in labour [113]. This supports priming of neutrophils in the periphery to facilitate migration to gestational tissue as part of the pathological processes of preterm parturition.

### 4.3. Foetal Membrane and Amniotic Fluid Neutrophils in Preterm Labour and PPROM

Chorioamnionitis, which refers to inflammation of the foetal membranes is observed in over 40% of cases of all preterm births, but as high as 94% in women who deliver between 21–24 weeks [79,114,115]. It is more commonly associated with PPROM, and with amniotic fluid cultures positive for bacteria [79]. Histological evidence of chorioamnionitis is characterised by maternal peripheral neutrophil infiltration of the chorioamnion. Several studies have confirmed that the most significant source of neutrophils is from the mother [80,116,117] although evidence also exists to suggest some contribution from the foetus [117]. The neutrophils migrate towards a chemotactic gradient, with in vitro evidence demonstrating the chemotactic effect of both unstimulated and LPS stimulated foetal membranes [118]. Conditioned media from LPS stimulated foetal membranes leads to the release of cytokines, chemokines, and reactive oxygen species from neutrophils, as well as neutrophil degranulation and NET release [118]. Additionally, explants from foetal membranes from both term [119] and preterm [120] pregnancies reveal complement activation, as well as presence of pro-inflammatory cytokines (IL-6, IL-8) and MMPs (MMP-9), all mediators influencing a pro-inflammatory milieu in foetal membranes, highly likely due to the local activity of neutrophils. Neutrophils are highly likely to play a predominant role in influencing the pro-inflammatory milieu in foetal membranes.

Amniotic fluid also contains inflammatory mediators and immune cells, that can be used clinically to establish the presence of intra-amniotic inflammation and infection. Amniotic fluid cytokines such as IL-8, IL-6, IL-1β [121], and complement proteins C5a and C3a [122,123] are increased in women who deliver preterm with or without intra-amniotic infection. Levels of MMP-8 [124] were higher in women with microbial invasion of the intra-amniotic cavity (MIAC) who ended up delivering preterm, compared to women who delivered at term. Neutrophil elastase was also reported in cases of PPROM, with even higher levels if they also had MIAC [125]. These immune mediators are likely to reflect activation of local neutrophils in response to ascending pathogens. Ex vivo studies demonstrate that amniotic fluid neutrophils can phagocytose bacteria typically associated with MIAC (*S. agalactiae*, *U. urealyticum*, *G. vaginalis*, *E. coli*) [126]. They are also likely to be able to perform NETosis, given that amniotic fluid neutrophils are surrounded by bacteria and a NET-like structure when taken from women with microbial culture positive amniotic fluid [127,128].

RNA sequencing of neutrophils and macrophages isolated from amniotic fluid from women with MIAC has revealed the cell types have different genes that are upregulated and downregulated [85], suggesting that even if both cell types are present, they are implicated in different roles. Neutrophils were enriched for “phagosome” and NOD-like receptor pathways. The same study compared the transcriptome of neutrophils retrieved by amniocentesis between women who deliver at term (n = 2) and preterm (n = 4). Although both clinical groups displayed an abundant inflammatory response (as determined by concentrations of IL-6 and white cell count), a greater transcriptional activity was observed in women who delivered preterm [85].

Funisitis, inflammation of the umbilical cord (including the umbilical artery, vein and Wharton’s jelly) reflects the foetal inflammatory response, and histology reveals infiltration of foetal-derived neutrophils [79]. The clinical counterpart is the foetal inflammatory syndrome (FIRS), which is characterised by high levels of levels of IL-6 in cord plasma and is correlated with worse neonatal outcomes [129].

### 4.4. Neutrophils and Cervical Remodelling in Preterm Term Labour

Inflammatory mediators in cervical-vaginal fluid (CVF) have frequently been reported to serve as potential biomarkers for the prediction of preterm birth [130]. The goal for vaginal microbial health is dominance of *Lactobacillus* spp. with *L. crispatus* particularly protective against adverse pregnancy outcomes (including PPROM, cervical shortening and sPTB) [101,102,131,132]. In contrast *Lactobacillus* depletion and high diversity vaginal microbiota [94,103,131,133], or dominance of *L. iners* [131] are associated with an increased risk of PPROM and sPTB. Dysbiosis and *Lactobacillus* depletion has been associated with higher concentration of CVF cytokines and chemokines (ICAM-1, IL-1β, GM-CSF, TNF-α, IL-8, IL-6) [102,103,104,134] proteases (MMP-1, MMP-8) [102,135], complement (C3b, C5) [104,136] and β defensin [137] in the context of preterm birth.

Many of these inflammatory mediators are chemoattractants or products of activated neutrophils. Neutrophils can be detected in vaginal fluid in cases of bacterial vaginosis [138,139] and they exhibit a higher expression of CD16 (Fc receptor) [139]. In term labour, neutrophil presence correlates with IL-8 levels [140]. In the context of PTB, their presence has been associated with granulocyte elastase [81], and with a higher vaginal pH and decreased presence of *lactobacilli* [141].

There is a paucity of studies examining the immunophenotype of neutrophils at the cervical-vaginal interface in the context of preterm labour, which is likely due to the challenges of sampling. A study by Hunter et al. sampled 120 women at high risk of preterm birth between 12–25 weeks and found that cervical neutrophils, although not always present, were the most abundant immune cell type at the cervical-vaginal interface [106]. Furthermore, in women with leukocytes present, higher concentrations of CVF IL-8, IL-6 and IL-1β were seen. No statistically significant differences were seen in neutrophil concentrations between women who delivered preterm compared to at term, however the numbers of preterm deliveries were small (n = 18). Mohd Zaki et al. have also reported that neutrophils are the predominant leukocyte population at the cervical-vaginal interface of women at high risk of preterm birth [105]. No significant differences were seen in neutrophil concentrations between women who delivered at term compared to preterm, however the study was likely to be underpowered for this outcome as only six women delivered preterm. RNA-seq was performed on cervical neutrophils from a subset of the cohort (n = 9). Despite small numbers, the expression of genes involved in neutrophil activation and degranulation negatively correlated with the presence of *G. vaginalis* and positively correlated with the presence of *L. iners* in matched vaginal swabs. We have shown that in women at high risk of preterm birth, neutrophils are more likely to be present at the cervical-vaginal interface if the microbial composition is one of high risk of preterm birth (CST III/*L. iners*, or CST IV, diverse), compared to low risk (CST I/*L. crispatus*, CST II/*L. gasseri*, CST V/*L. jensenii*) [107]. Furthermore, in women that have detectable live cervical neutrophils, there are higher concentrations of pro-inflammatory mediators such as C3b, C1q and C4b in the cervical-vaginal fluid. Taken together, these data suggest a plausible role for cervical neutrophils in microbial driven cervical shortening and PTB [107].

## 5. Conclusions

Neutrophils are polymorphonuclear cells and are the most predominant circulating innate immune cells. They play a key role in both inflammation and infection, with effector functions that lead to direct and indirect cell death and microbial clearance. Activated neutrophils secrete pro-inflammatory mediators such as cytokines, proteases and collagenases, and pro-labour mediators such as COX-2 and PGE2. These mediators are required for the physiological processes of healthy term labour; cervical remodelling, uterine contractility, and foetal membrane rupture. However, in cases of microbial driven preterm birth, the premature recruitment of neutrophils into the cervix, uterus and foetal membranes, combined with increased activation, are likely to play a key role in triggering PPROM and PTB. An improved understanding of the functional role of neutrophils in the pathophysiology of preterm labour could lead to the development of novel predictive tools and therapeutic strategies.

## Figures and Tables

**Table 1:** Content of neutrophil granules and secretory vesicles.

Function	Primary/ Azurophilic	Secondary/ Specific	Tertiary/ Gelatinase	Secretory Vesicles
Microbicidal	Myeloperoxidase Lysozyme Defensins Cationic proteins Azurocidin Cap57	Lysozyme Lactoferrin Pentraxin 3 Lipocalin 2 Haptoglobin	Cathelicidin Lysozyme	
Serine proteases	Neutrophil elastase Cathepsins			
Metalloproteinases	Proteinases	Collagenase (MMP-8)	Gelatinase B (MMP-9) Leukolysin (MMP-25) Collagenase (MMP-8)	Leukolysin (MMP-25) Proteinase 3 (myeloblastin)
Acid hydrolases	Cathepsins B β-glucuronidase Glycerophosphatase N-acetylglucosaminidase α-mannosidase		β2-microglobulin	
Others	Heparin binding protein (HBP) Sialidase Presenilin Granulophysin α1-antitrypsin	Histaminase Heparanase Stomatin B12 binding protein Cytochrome b C3b receptor	C3bi receptor	Heparin-binding protein (HBP) Plasma proteins (including albumin) Alkaline phosphatase

## Data Availability

No new data were created or analysed in this study. Data sharing is not applicable to this article.
